# Radial Growth and Wood Density Reflect the Impacts and Susceptibility to Defoliation by Gypsy Moth and Climate in Radiata Pine

**DOI:** 10.3389/fpls.2018.01582

**Published:** 2018-10-31

**Authors:** Jesús Julio Camarero, Flor Álvarez-Taboada, Andrea Hevia, Fernando Castedo-Dorado

**Affiliations:** ^1^Instituto Pirenaico de Ecología, Consejo Superior de Investigaciones Científicas (IPE-CSIC), Zaragoza, Spain; ^2^Grupo de Investigación en Geomática e Ingeniería Cartográfica, Universidad de León (de Ponferrada), León, Spain; ^3^Forest and Wood Technology Research Centre (CETEMAS), Siero, Spain; ^4^Departamento de Ciencias Agroforestales, Universidad de Huelva, Huelva, Spain

**Keywords:** *Castanea sativa*, dendroecology, extreme climate event, defoliation, intra-annual density fluctuation, *Lymantria dispar dispar*, *Pinus radiata*, wood density

## Abstract

Drought stress causes a reduction in tree growth and forest productivity, which could be aggravated by climate warming and defoliation due to moth outbreaks. We investigate how European gypsy moth (*Lymantria dispar dispar* L., Lepidoptera: Erebidae) outbreak and related climate conditions affected growth and wood features in host and non-host tree species in north-western Spain. There, radiata pine (*Pinus radiata* D. Don) plantations and chestnut (*Castanea sativa* Mill.) stands were defoliated by the moth larvae, whereas Maritime pine (*Pinus pinaster* Ait.) was not defoliated. The gypsy moth outbreak peaked in 2012 and 2013, and it was preceded by very warm spring conditions in 2011 and a dry-warm 2011–2012 winter. Using dendrochronology we compared growth responses to climate and defoliation of host species (radiata pine, chestnut) with the non-host species (Maritime pine). We also analyzed wood density derived from X-ray densitometry in defoliated and non-defoliated trees of radiata pine. We aimed to: (i) disentangle the relative effects of defoliation and climate stress on radial growth, and (ii) characterize defoliated trees of radiata pine according to their wood features (ring-width, maximum and minimum density). Radial growth during the outbreak (2012–2013) decreased on average 74% in defoliated (>50% of leaf area removed) trees of radiata pine, 43% in defoliated trees of chestnut, and 4% in non-defoliated trees of Maritime pine. After applying a BACI (Before-After-Control-Impact) type analysis, we concluded that the difference in the pattern of radial growth before and during the defoliation event was more likely due to the differences in climate between these two periods. Radiata pines produced abundant latewood intra-annual density fluctuations in 2006 and 2009 in response to wet summer conditions, suggesting a high climatic responsiveness. Minimum wood density was lower in defoliated than in non-defoliated trees of radiata pine prior to the outbreak, but increased during the outbreak. The pre-outbreak difference in minimum wood density suggests that the trees most affected by the outbreak produced tracheids with wider lumen and were more susceptible to drought stress. Results of this study illustrate (i) that the pattern of radial growth alone may be not a good indicator for reconstructing past defoliation events and (ii) that wood variables are reliable indicators for assessing the susceptibility of radiata pine to defoliation by the gypsy moth.

## Introduction

In the Anthropocene, forests face unprecedented pest problems that are outside managers' range of experience (Liebhold, 2012). Outbreaks of herbivorous insects causing widespread disturbances may be favored by climate warming, land-use changes, increasing host abundance or susceptibility and range shifts of pests (Raffa et al., 2008). There may also be feedbacks between components of the changes at the global scale which may aggravate pest incidence (Ayres and Lombardero, 2018). Tools to quantify how susceptible trees are to pests and to monitor effects of pests on forest growth and health will help managers to develop strategies to face new challenges (Trumbore et al., 2015).

Rapid land-use changes during the twentieth century have resulted in millions of hectares of pine plantations, particularly in areas in the southern Hemisphere where pines are introduced species (FAO, 2010). These commercial plantations involve also biota alterations because they are monocultures of non-native tree species which may face new herbivores (Wingfield et al., 2015). In some cases, these plantations can reduce herbivory relative to their native environments via geographical escape from their natural herbivores. However, sometimes the native herbivores can become important pests of non-native tree species (Nuñez et al., 2008).

Outbreaks of defoliating insect herbivores may reduce productivity in commercial plantations, such as those of the widely planted radiata pine (*Pinus radiata* D. Don), because they reduce stand productivity. Radiata pine is native to California but has been extensively used for commercial forestry, becoming the most extensively planted softwood worldwide (Mead, 2013). More than four million ha of radiata pine plantations exist, and 90% of them are located in the southern Hemisphere (Manley and Maclaren, 2009). In this regard, we need an improved monitoring of the responses of radiata pine in plantations to insect defoliators which may make their management challenged by the changes at the global scale described above.

The European gypsy moth (*Lymantria dispar dispar* L., Lepidoptera: Erebidae) is a problematic defoliating pest of angiosperm and gymnosperm tree species in the northern Hemisphere because it is a polyphagous species (Tobin and Liebhold, 2011). In north-eastern USA the gypsy moth is invasive, and outbreaks have been observed throughout the twentieth century, differently affecting tree species indicating that tree species vary in their susceptibility to defoliation (Allstadt et al., 2013). Radiata pine has been conventionally considered resistant to defoliation, i.e., needles are consumed only by some larval stages or when susceptible hardwood species (consumed by all larval stages) are not present (Liebhold et al., 1995).

In the laboratory it was demonstrated that European gypsy moth could complete development to adult by feeding on needles of radiata pine (Miller and Hanson, 1989). Moreover, the outbreak in the present study showed the ability of this insect to complete development in the field and severely defoliated trees of radiata pine (Castedo-Dorado et al., 2016).

Here, we capitalize on the outbreak reported by Castedo-Dorado et al. (2016) which mainly affected plantations of radiata pine but also caused defoliation in the native chestnut (*Castanea sativa* Mill.) but not in planted Maritime pine (*Pinus pinaster* Ait.) trees in northern Spain (Lago-Parra et al., 2016). An increase in gypsy moth populations was first noticed in 2011, peaking in 2012 and 2013 (maximum defoliation severity) and then insect populations collapsed in 2014 (Castedo-Dorado et al., 2016). We used tree-ring data to evaluate the impacts of the outbreak and related climate anomalies (i.e., 2011 and 2012 were very dry and warm years over most northern Spain; cf. Camarero et al., 2015) on radial growth of susceptible (i.e., radiata pine, chestnut) and immune or non-host species (e.g., Maritime pine). Since wood density is a key characteristic for the timber industry, we also quantified changes in maximum and minimum wood density of defoliated and non-defoliated trees of radiata pine.

Dendroecology has been used to evaluate the effect of insect defoliations on radial growth by comparing tree-ring width series of host vs. non-host species (Swetnam et al., 1985). Narrow rings or morphological wood anomalies (e.g., xylem cells with poorly lignified walls) have been also associated with defoliations (Sutton and Tardif, 2005, 2007; Paritsis et al., 2009). Densitometry has also been used to characterize the reduction in maximum wood density caused by some outbreaks (Esper et al., 2007; Koprowski and Duncker, 2012).

In this study, (i) we assessed the relative effects of gypsy moth defoliation and climate on radial growth and wood density, in the case of radiata pine, and (ii) we compared the responses of susceptible or host (chestnut, radiata pine) vs. immune or non-host (Maritime pine) tree species to this outbreak. We expected that insect defoliation to result in a severe loss of radial growth and reduction in wood density in radiata pine trees, whereas warm-dry conditions leading to water scarcity to result in growth reduction in non-defoliated trees of radiata pine and in immune trees of Maritime pine.

## Materials and methods

### Study area and tree species

The gypsy moth outbreak occurred in the Municipality of Cubillos del Sil, province of León, north-western Spain (42° 41' N, 6° 36' W). According to the records of the Office responsible for forest pest control in the region (Junta de Castilla y León), it was the first record of outbreak by the gypsy moth in the area since plantation establishment in the 1990s. The mean altitude of the study area is 570 m a.s.l. The stand is facing east and has a slight slope (5–10°). Climate in the study area is Mediterranean temperate with oceanic influence. According to climatic data for the period 1971–2015 taken from the Ponferrada climatic station (42° 33' N, 6° 35' W, 534 m a.s.l.), located at about 12 km from the study site, the mean annual temperature is 12.7°C, with a mean daily minimum of 0.9°C in January and a mean daily maximum of 29.1°C in July. The mean annual rainfall is around 672 mm (Figure S1a). The soils are acidic and low in nutrient concentrations. Vegetation in the area is dominated by radiata pine plantations, although stands of native broadleaved species are also present, including chestnut and oak species (*Quercus ilex* L., *Quercus pyrenaica* Willd). Maritime pine occurred in clumps within radiata pine stands, and originated from natural regeneration from seeds produced by trees in the previous rotation.

The outbreak mainly affected a pure stand of radiata pine planted in 1994 (trees were 18 years old at the beginning of the outbreak in 2012). The site index of the forest was around 20 m (according to the height growth curves of Diéguez-Aranda et al., 2005). Density at establishment was ~1,600 trees ha^−1^, which was reduced to ~800 trees ha^−1^ by a thinning of under-performing trees in early 2012.

**Figure 1:**
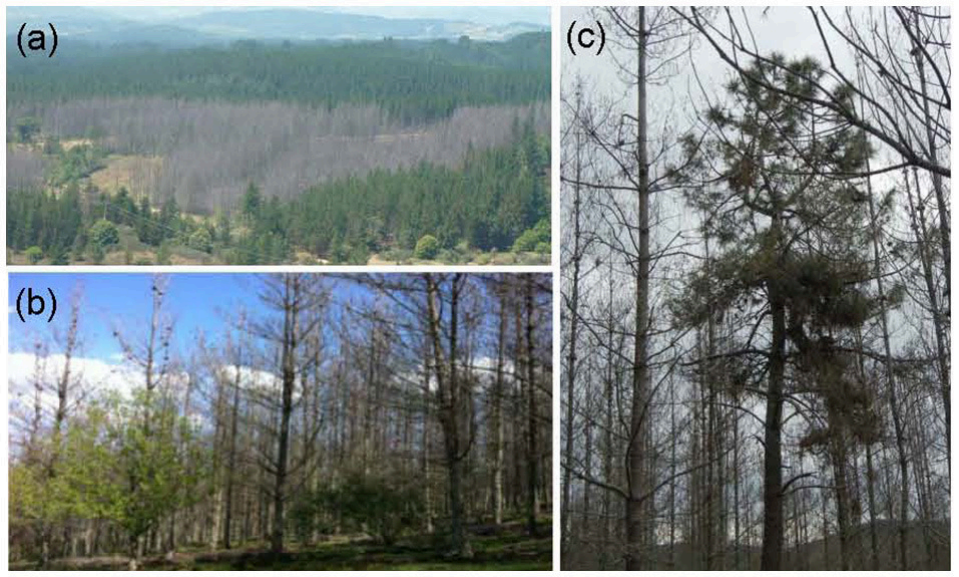
Some illustrative views of the defoliation caused by the gypsy moth outbreak in the studied area (Cubillos del Sil, north-western Spain). **(a)** Landscape view of radiata pine affected plantations, **(b)** defoliated radiata pine trees and chestnut stands, and **(c)** defoliated radiata pines surrounding a Maritime pine not affected by defoliation.

After the defoliation event of 2012–2013, *ca*. 6 ha were severely defoliated during two consecutive years (2012 and 2013), and *ca*. 40 ha were severely defoliated in 2012. Moderate defoliation was observed in surrounding stands of radiata pine affecting *ca*. 15 ha. Virtually all noticeable defoliation was limited to radiata pine (Figure 1a), except a small chestnut stand of *ca*. 1 ha, located within a radiata pine stand (Figure 1b). Isolated Maritime pines were not affected indicating a different susceptibility to the gypsy moth of the two pine species (Figure 1c).

### Field sampling and dendrochronology

In April 2014, at the end of the outbreak, we selected and labeled 46 dominant *P. radiata* trees (18 defoliated and 28 non-defoliated trees; see Figure S1b). A threshold of 50% of loss in leaf area was used to classify trees as defoliated or non-defoliated individuals. Defoliation was noted in the field by visual assessment and, when necessary, using binoculars. The defoliation class assigned to each tree at the time of the defoliation assessment (April 2014) was assumed to be the same as during the defoliation period (2012–2013).

In January 2016, all the selected radiata pine trees, along with 21 chestnut trees, and 13 Maritime pine trees were sampled for dendrochronological analyses. The 13 Maritime pine trees and the 11 defoliated trees of chestnut were randomly selected in the vicinity of sampled radiata pine trees (Figure S1b). The remaining 10 chestnut trees were sampled in a nearby stand where defoliation did not occur. All the sampled trees of the three species were georeferenced using a sub-meter GPS receiver. Given that the study area is quite homogenous regarding topography and soil type, selected trees for each species and defoliation class were very similar in size, and therefore, we consider this number of trees sufficient to characterize the radial-growth patterns.

Tree diameter at breast height (dbh) was measured at 1.3 m for each tree using tapes. Sampling was performed following standard dendrochronological methods (Fritts, 2001). Two cores were taken at 1.3 m using a 5 mm Pressler increment borer from the 80 sampled trees. The wood samples were air-dried and polished with successively finer sand-paper grits until rings were clearly visible. Then, wood samples were visually cross-dated.

In radiata pine, we quantified (percent annual frequency) intra-annual density fluctuations (IADFs) in the latewood which reflect the tree responsiveness to intra-annual variability in precipitation (Pacheco et al., 2018). Latewood IADFs are characterized by the presence of earlywood-like cells, i.e., having wider lumen and thinner cell walls than latewood tracheids (Vieira et al., 2010). Latewood IADFs were visually identified in all cross-dated sampled under a binocular microscope.

Tree-ring width was measured to the nearest 0.001 mm with a stereomicroscope mounted above a LINTAB System (Rinntech, Heidelberg, Germany) device linked to a computer. Cross-dating of the tree rings was carried out using the program COFECHA (Holmes, 1983). The trend due to the geometrical constraint of adding a volume of wood to a stem of increasing radius was corrected by converting tree ring widths into basal area increment, hereafter abbreviated as BAI, which is a more biologically meaningful descriptor of growth trends than ring widths (Biondi and Qeadan, 2008). Annual basal area increment was calculated from tree-ring widths as the difference between consecutive basal areas using the following expression:

(1) $$\begin{array}{r} \text{BAI}=\pi \left(r_{t}^{2}-r_{t-1}^{2}\right) \end{array}$$

where BAI is the basal area increment and *r*_*t*_ and *r*_*t*__−1_ are the radii corresponding to rings formed in years *t* and *t*-1, respectively.

### Densitometry analyses

For the densitometry analyses, we additionally sampled 20 radiata pine trees, 10 defoliated and 10 non-defoliated. Trees were sampled at 1.3 m using a 12 mm Pressler increment borer. Wood samples were air dried and glued onto wooden mounts in the laboratory. For each core, thin laths of 1.2 mm thickness were cut by a twin bladed saw. The laths were then x-rayed in an Itrax Multiscanner (Cox Analytical Systems, Sweeden) at the CETEMAS laboratory (Asturias, Spain). The Multiscanner, equipped with a Cu-tube (Bergsten et al., 2001), was operated at 30KV and 50 mA, with an exposure of 25 s at each sample point and a step size of 20 μm (Moreno-Fernández et al., 2018).

The radiographic images obtained from the Itrax Multiscanner were analyzed in WinDENDRO (Regent Instruments, Canada) to determine wood density values for each point in the wood sample. This allowed us to define wood density profiles and to determine maximum and minimum wood density values for each annual ring. Cross-dating was again carried out by using the COFECHA software (Holmes, 1983).

### Statistical analyses

Comparisons of variables of interest (BAI and wood density) between defoliated and non-defoliated trees of the same species were assessed using the Mann-Whitney *U*-test.

To perform climate-growth correlations in non-defoliated trees, tree-ring width data were converted into ring-width indices to remove any size- or age-related growth trend (Cook et al., 1990). First, we removed the long–term trends of tree-ring width series by detrending them using negative exponential functions, which preserve high-frequency variability in the indices. Residuals were obtained by subtracting observed tree-ring width data from fitted values. Second, autoregressive models were fitted to each detrended series to remove most of the first-order autocorrelation. Lastly, a bi-weight robust mean was used to obtain chronologies for each species. A similar procedure was used for maximum and minimum wood density data of radiata pine but in this case indices were calculated as ratios from the fitted growth curves. Chronology development was done using the ARSTAN program (Cook and Krusic, 2005).

Using the ring-width indices obtained as described in the previous paragraph, the climate-growth correlations were calculated for the common and best-replicated period 2000–2015 considering only non-defoliated trees (Fritts, 2001). Tree radial growth was described by averaged annual ring-width indices whereas climatic variables (mean of the maximum and mean of the minimum temperatures, precipitation) were calculated annually from the Ponferrada station. Climatic variables were calculated for each month and season (winter, previous December to February; spring, March to May; summer, June to August; autumn, September to November). Mean of the maximum and mean of the minimum temperatures refers to the mean of the daily maximum and minimum temperature, respectively, for a given period. The window of analyses included from the previous October to current September based on previous studies (Downes et al., 1993; Ivković et al., 2013). The correlation analyses of the 16 climate-growth data pairs were carried out using the Pearson correlation coefficient. This correlation coefficient was also used for assessing the climate-wood density correlations for the period 2000–2015 for both defoliated and non-defoliated trees. We did not remove temporal autocorrelation of climate data since most of the considered variables it variables was not significant (*P* > 0.05) at 1–10 lags (Table S1).

For disentangling the effect of climate and defoliation on radial growth, we applied the BACI (Before-After-Control-Impact) type concept. As we have available information prior and subsequent to a stressor (defoliation), and information of stressed (defoliated) and control (non-defoliated) entities (trees), our data can be viewed as a case of a BACI design. The method for analysis this type of data involve comparison of stressed entities with control entities, and before and after they were affected by the stressor (e.g., Green, 1979; Stewart-Oaten et al., 1986).

Following this BACI-type concept we fitted a linear mixed-effects model of BAI of the current year (BAI_*t*_) for a given tree as a function of climate data of the current growing season (or of the previous growing season), the defoliation class (defoliated or non-defoliated), the type of period (before defoliation or during the defoliation) and the interactions among them. The whole period 2000–2013 was considered because exploratory analysis of the data showed that the different length of the time series before the defoliation event (12 years) and that during the event (2 years) did not condition the results. Unlike in the correlation analysis described earlier, the BAI data used in the linear mixed model were not detrended.

For radiata pine and chestnut trees, the following full model was fitted:

(2) $$\begin{array}{rcl} y & = & \;b_{0}+\;b_{1}x_{1}+b_{2}x_{2}\;+b_{3}x_{3}+b_{12}x_{12}+b_{13}x_{13}+b_{23}x_{23}\\ +b_{123}x_{123}+d\;z+\;\varepsilon \end{array}$$

where *y* = response variable (annual BAI_*t*_ for a given tree), *b*_0_ = intercept; *x*_1_ = climate covariate, *x*_2_ = an indicator variable distinguishing between defoliated and non-defoliated trees, *x*_3_ = an indicator variable distinguishing between before defoliation and during the defoliation period, *x*_12_ = interaction between climate and defoliation, *x*_13_ = interaction between climate and period, *x*_23_ = interaction between defoliation and period, *x*_123_ = interaction among climate, defoliation and period, *z* = tree identity (the random effect); *b*_1_, *b*_2_, *b*_3_, *b*_12_, *b*_13_, *b*_23_, and *b*_123_ = coefficients for the above-mentioned covariate, fixed-effects factors, or interaction terms; *d* = coefficients for the random effect; and ε = error term, which was modeled using a first-order autoregressive correlation structure [AR(1)] for dealing with the temporal autocorrelation of the BAI_t_ data.

Nested reduced models considering a three two-way interactions (one model), two two-way interactions (three models) and only one two-way interaction (three models) were also tested for assessing the impact of subtracting predictors from the full model.

For Maritime pine trees, which did not suffer defoliation, the full mixed model fitted was the following:

(3) $$\begin{array}{r} y=\;b_{0}+\;b_{1}x_{1}\;+b_{3}x_{3}+b_{13}x_{13}+d\;z+\;\varepsilon \end{array}$$

The nested reduced model for this species did not considered the interaction term.

For all the models, BAI_*t*_ was log-transformed to meet the assumption of normality of the data.

Seasonal climate variables were used to avoid fitting too complex models based on monthly climate variables. It must be noted that the climate data may also be correlated at short to long time scales, but the possible autocorrelation in the climate data is not accounted in the models since previous analyses revealed that the climate series did not show significant temporal autocorrelation at 1–10 years scales (Table S1).

Comparisons between models were carried out through the log-likelihood ratio statistic (-2LL), which follows a chi-square distribution with degrees of freedom equal to the difference between the number of coefficients of the models (Zuur et al., 2009). The random effects and the covariance parameters were estimated using the maximum-likelihood method (Zuur et al., 2009) implemented in the lme function of the nlme library in R (R Core Team, 2017).

Taking into account that differences in tree radial growth can arise because of different microsite conditions (e.g., access to soil water), and the experimental design did no factor out microsite differences, we examined whether spatially autocorrelated factors, such as microsite differences, were present. Using ArcGIS® software (ESRI, 2011) we calculated Moran's I index for average BAI of both the non-defoliation and the defoliation periods. This index evaluates whether the pattern is spatially autocorrelated or not (random; Goodchild, 1986).

## Results

### The 2011–2012 climate anomalies

The outbreak was preceded by very warm spring conditions in 2011 (+6.0° C anomaly in the mean of the maximum April temperatures; warmest on record since 1971) and a dry 2011–2012 winter (Figures S2,S3). The 2 years of the gypsy moth outbreak culmination (2012 and 2013) were characterized by very dry conditions, particularly in early 2012 (−50 mm anomaly in February precipitation; driest February on record), and were comparable in terms of water shortage with other years characterized by dry spells as 2005 (Figure S3).

### Tree size, radial growth, IADFs, and wood density: comparisons of defoliated and non-defoliated trees within each species

**Figure 2:**
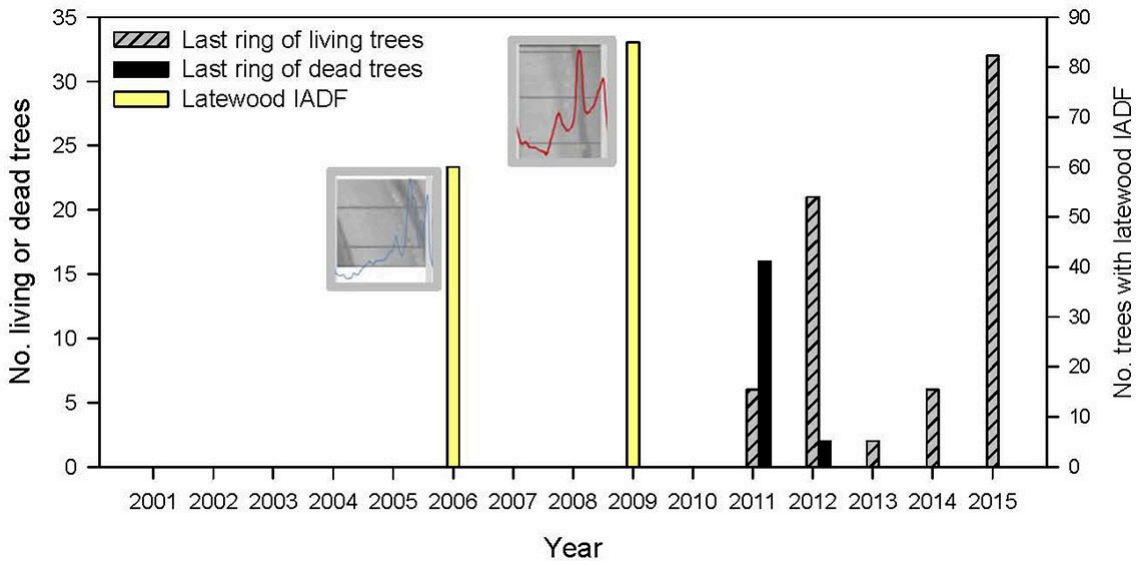
Number of living or dead radiata pine trees according to their last formed tree-ring (left *y* axis) or according to the presence of intra-annual density fluctuations (IADF) in the latewood (right *y* axis). The insets show density profiles and images corresponding to the 2006 and 2009 rings when latewood IADFs were abundant. Dead pines died during the gypsy moth outbreak.

The dbh of defoliated and non-defoliated trees were similar in radiata pine and chestnut (Table 1). According to the cross-dated ring-width series of defoliated trees of radiata pine, most of them (89%) formed the last complete ring in 2011, whereas 33 and 47% of living trees formed it in 2012 and 2015, respectively (Figure 2). This means that many severely defoliated trees did not form a complete ring in 2012, when defoliation peaked, whilst other defoliated trees of radiata pine presented extreme growth reduction in 2012 and stopped growing. Two years (2006, 2009) presented abundant IADFs in the latewood of radiata pine trees (Figure 2). Using 2009 to illustrate the climate conditions linked to IADF formation, these corresponded to warm conditions but mainly elevated summer (June to July) and autumn-winter (November-December) precipitation (Figure S3).

**Table 1:** Size (diameter at breast height, dbh) and radial-growth data (basal area increment, BAI) measured for the sampled tree species.

**Species**	**Tree status**	**No. trees**	**dbh (cm)**				**BAI 2000–2011 (cm^2^)**				**BAI 2012-2013 (cm^2^)**			
			**Max**	**Min**	**Mean**	**SE**	**Max**	**Min**	**Mean**	**SE**	**Max**	**Min**	**Mean**	**SE**
*Pinus radiata*	Defol	18	21.3	16.4	18.7	1.8	20.49	4.61	13.11a	1.20	10.43	1.26	2.95a	0.87
	Non-defol	28	24.6	18.2	21.0	2.1	21.64	5.48	16.89b	1.28	28.45	1.52	11.28b	2.32
*Castanea sativa*	Defol	11	20.5	16.7	18.4	0.4	14.09	6.11	9.71a	1.40	12.66	5.92	8.96a	0.43
	Non-defol	10	20.0	16.9	18.2	0.3	14.56	7.07	10.11a	1.44	25.06	7.63	15.66b	0.55
*Pinus pinaster*	Non-defol	13	32.2	24.9	28.5	2.6	42.41	7.99	20.21	1.21	38.51	1.84	19.39	4.29

*Basal area increment was calculated for the period previous to defoliation (2000–2011) and for the outbreak period (2012–2013). Different letters indicate significant BAI differences (α = 0.05) between defoliation classes (defoliated –Defol–, and non-defoliated –Non-defol) within each species (radiata pine and chestnut) based on Mann-Whitney U-tests. Values are means, maximum (Max), and minimum (Min) values, and standard errors (SE)*.

**Figure 3:**
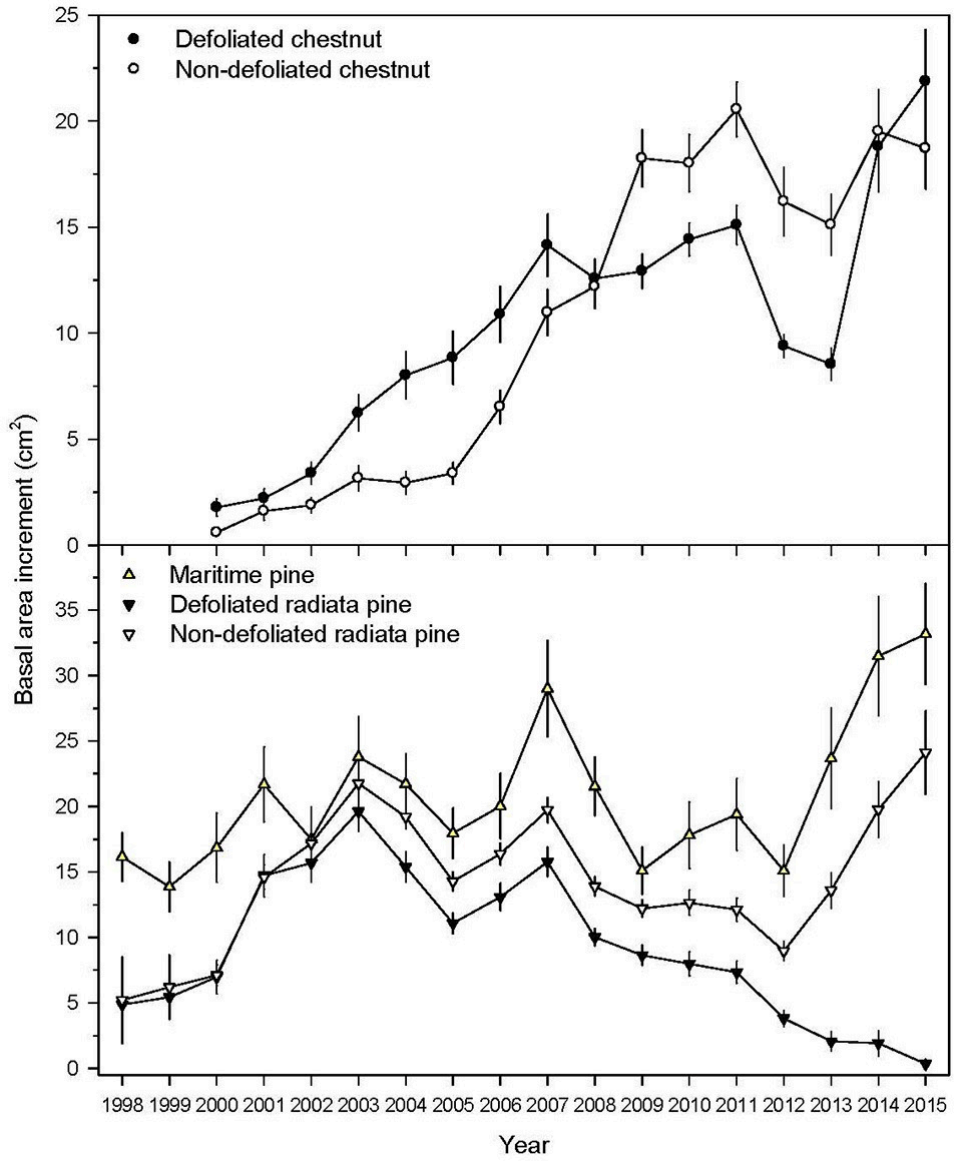
Growth patterns (basal area increment) of the three studied tree species and considering defoliated and non-defoliated trees due to the gypsy moth outbreak in the case of chestnut and radiata pine. Values are means ± SE.

Prior to the outbreak (period 2000–2011), we found no significant differences in BAI between defoliated and non-defoliated trees of chestnut, but defoliated trees of radiata pine showed a lower BAI than their non-defoliated counterparts (*U* = 64, *P* < 0.001; Table 1 and Figure 3). Defoliated trees of radiata pine and chestnut showed significantly lower BAI values during the outbreak (2012–2013) than their non-defoliated conspecifics (Table 1 and Figure 3). The mean BAI values of the defoliated trees of radiata pine continue to decrease during and after the defoliation, while that of the non-defoliated trees of radiata pine continue to increase during the same period (2012–2015; see Figure 3). The temporal pattern of changes in the mean BAI values of Maritime pine trees was similar to that of non-defoliated trees of radiata pine. In contrast to the radiata pine, the mean BAI of the defoliated trees of chestnut showed an increasing trend after the defoliation (2014–2015; see Figure 3). The mean reductions of BAI in defoliated trees during 2012–2013 compared with 2000–2011 were 74% and 43% in radiata pine and chestnut, respectively. For immune maritime pines (i.e., non-defoliated) BAI declined 4% during the outbreak period compared with the pre-outbreak period.

**Figure 4:**
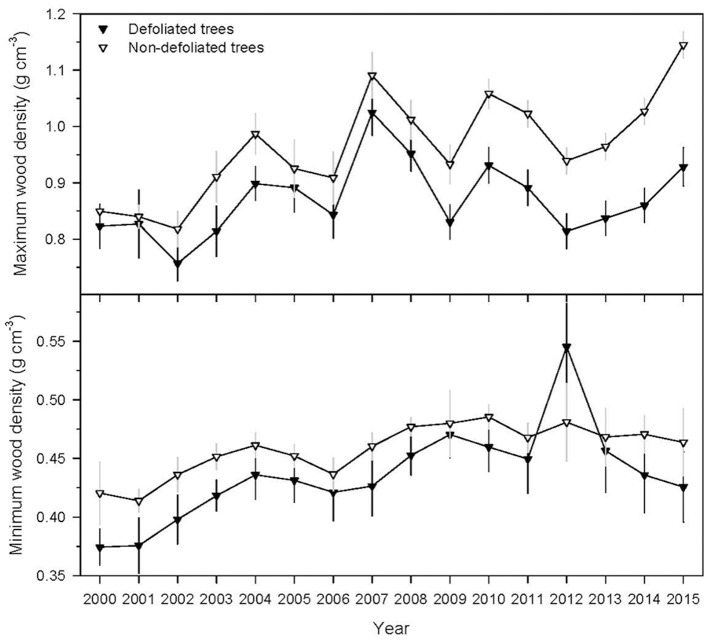
Maximum and minimum wood density of defoliated and non-defoliated radiata pines. Values are means ± SE.

Prior to the outbreak, defoliated trees of radiata pine had lower mean maximum wood density (mean ± SE = 0.87 ± 0.02 g cm^−3^) than non-defoliated (0.96 ± 0.02 g cm^−3^) pines (*U* = 50, *P* = 0.003). Similarly, defoliated trees presented lower mean minimum wood density (0.44 ± 0.01 g cm^−3^) than non-defoliated (0.46 ± 0.01 g cm^−3^) trees (*U* = 63, *P* = 0.015). However, in the year when outbreak peaked (2012), defoliated pines showed higher mean minimum wood density (0.54 ± 0.03 g cm^−3^) than non-defoliated (0.48 ± 0.02 g cm^−3^) pines (*U* = 20, *P* = 0.003) (Figure 4).

### Climate and defoliation effects on radial growth

**Figure 5:**
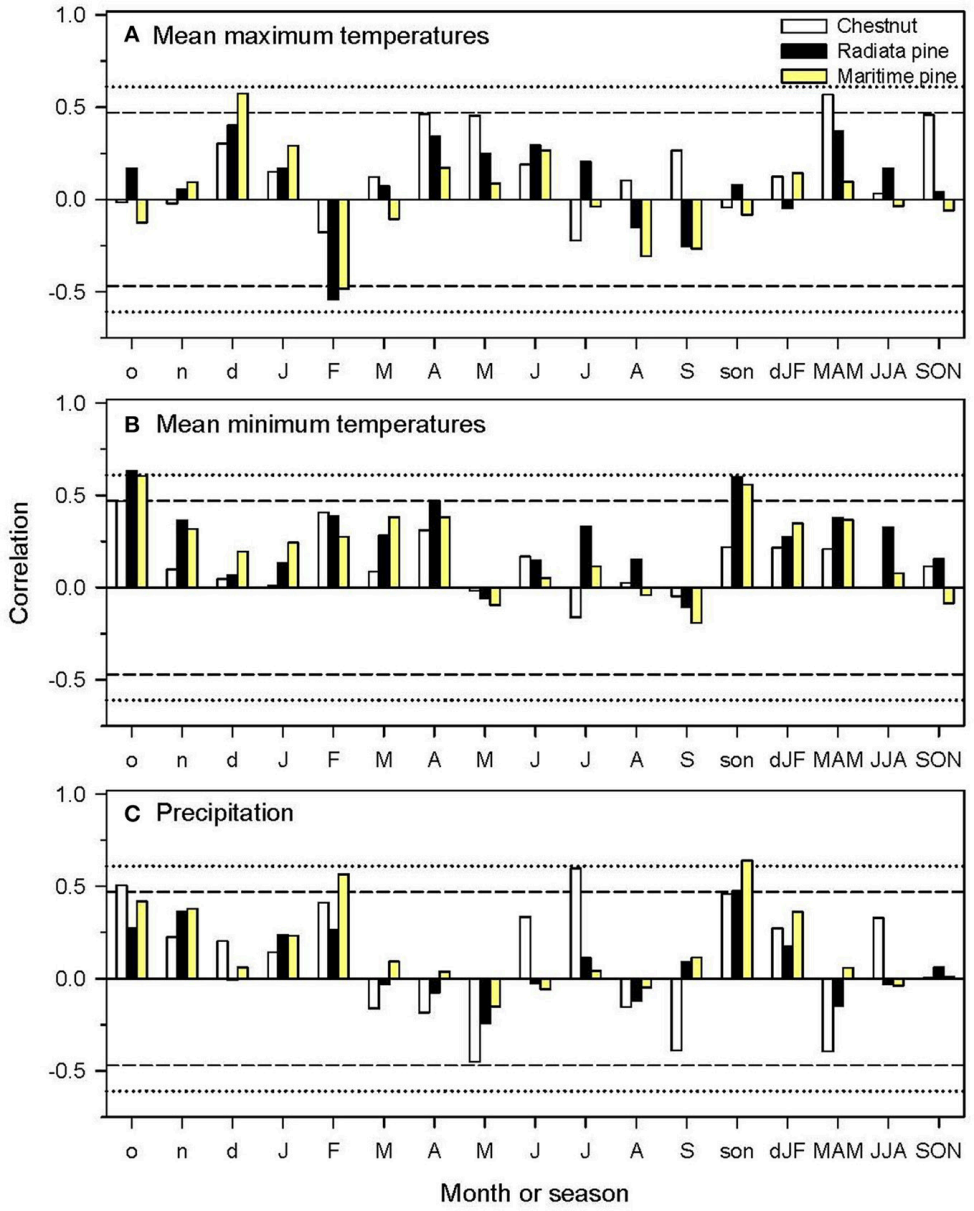
Climate-growth relationships calculated by relating monthly or seasonal climatic variables [**(A)**, mean maximum temperatures; **(B)**, mean minimum temperatures; **(C)**, precipitation] with the mean series of ring-width indices of the three study species (chestnut, radiata pine, maritime pine). The dashed and dotted horizontal lines correspond to the 0.05 and 0.01 significance levels, respectively. Months or seasons abbreviated by lowercase or uppercase letters correspond to the previous and current years, respectively.

The mean BAI series of radiata pine and Maritine pine for the 2000–2011 period were highly correlated (*r* = 0.68, *P* < 0.0001), but the chestnut BAI series was neither related to the radiata (*r* = −0.27, *P* = 0.39) nor to the Maritime pine BAI series (*r* = −0.007, *P* = 0.98). Considering the ring-width indices (plotted in Figure S4), we found that high mean maximum temperatures during the previous December were positively related to Maritime pine growth, and high mean minimum temperatures during the previous autumn were associated with improved growth in the two pine species (Figure 5). However, high maximum February temperatures were negatively related to growth of both pine species. Warm spring temperature favored chestnut growth. Wet conditions during the previous autumn were positively related to growth of the three species, whereas wet February and July conditions favored Maritime pine and chestnut growth, respectively.

The climate variables selected in the linear mixed-effects models are in agreement with the climate-growth correlations observed in Figure 5. According to these results, annual BAI was affected by prior minimum autumn temperatures in the case of the two pine species and by maximum spring temperatures in the case of chestnut.

For radiata pine, the three-way interaction (defoliation × climate × period) was not significant (*P* = 0.55). Moreover, all the two-way interactions were not significant (*P*-values ranging from 0.44 to 0.66), except the climate × period (*P* < 0.001 in all cases; Table S2 and Figure S5). The model containing this interaction was selected as the best according to the likelihood ratio test (Table 2). For Maritime pine, the full model (including the climate × period interaction) was also selected as the best (Tables 2, S2). For chestnut, none of the interaction terms was significant, and none of the models could be considered as the best according to the likelihood ratio test. Since none of the interaction terms was significant, we tested the significance of the main factors one at a time. The results indicate that both the climate variable (Tmax spring_*t*_) and the period were significant (Table 2).

**Table 2:** Parameter estimates and their corresponding *P*-values of the best linear mixed-effects models fitted to annual basal area increment (BAI_*t*_) for *Pinus radiata, Pinus pinaster* and *Castanae sativa*.

**Term**	***Pinus radiata***		***Pinus pinaster***		***Castanea sativa***	
	**Parameter est**.	***P*-value**	**Parameter est**.	***P*-value**	**Parameter est**.	***P*-value**
Intercept	34.69	<0.0001	37.14	<0.0001	–	–
Tmin autumn_t−1_	−3.562	<0.0001	−3.813	<0.0001	–	–
Tmax spring*_*t*_*	–	–	–	–	0.07918	<0.0001
Defoliation	−0.3825	0.02	–	–	−0.06078	0.91
Period	−28.26	<0.0001	−30.40	<0.0001	0.3110	0.007
Tmin autumn_t−1_ × Period	3.624	<0.0001	3.885	<0.0001	–	–
Phi	0.85		0.89		–

*For Castanea sativa the parameter estimates and P-values come from fitting a linear mixed-effects model using each covariate or factor one at a time. Phi is the autocorrelation parameter for the AR(1) error structure*.

Concerning the influence of climate and defoliation in wood density, it was observed that in non-defoliated trees of radiata pine, minimum wood density was positively associated with June precipitation (*r* = 0.61, *P* = 0.012) but negatively associated with March minimum temperatures (*r* = −0.64, *P* = 0.008). In defoliated trees of radiata pine, minimum wood density decreased as minimum April temperatures increased (*r* = −0.52, *P* = 0.040). High September minimum temperatures were negatively associated with maximum wood density in defoliated (*r* = −0.60, *P* = 0.014) and non-defoliated trees (*r* = −0.53, *P* = 0.038).

Average BAI prior to the outbreak or during the outbreak was not spatially autocorrelated for any of the three tree species, since the *P*-values were not statistically significant (*P* > 0.05 in all cases; Table 3) associated with Moran's I index.

**Table 3:** Values of the Moran's *I* index values used to test for the presence of spatial autocorrelation in growth data (BAI, basal area increment).

**Species**	**BAI 2000-2011**			**BAI 2012-2013**		
	**Moran's *I***	***Z* score**	***P-*value**	**Moran's *I***	***Z* score**	***P-*value**
*Pinus radiata*	0.0668	0.7112	0.48	0.1900	1.5792	0.11
*Castanea sativa*	−0.0984	−0.2910	0.77	0.1239	1.0792	0.28
*Pinus pinaster*	−0.2358	−1.4302	0.15	−0.2022	−1.1159	0.26

*Z scores and P-values evaluate the significance of the index. Analysis was carried out for the period previous to defoliation (2000–2011) and for the outbreak period (2012–2013)*.

## Discussion

For radiata pine, the absence of significance for the three-way interaction (defoliation × climate × period) indicates that the interactive effects of climate and defoliation on growth did not differ before and during the defoliation event. The significance of climate × period interaction highlights that the effect of climate differ before and during the defoliation event, i.e., the climate affected radial growth in a different manner in the two different periods. The absence of significance for the climate × defoliation interaction states that the effect of climate on growth did not differ between the defoliated and non-defoliated trees, whereas in the case for the defoliation × period interaction points out that the effect of defoliation did not differ before and during the defoliation event.

The fact that the two way-interaction terms involving defoliation were not significant suggests that, for radiata pine, the difference in the pattern of radial growth before and during the defoliation event (74%) was more likely due to the difference in climate before and during the defoliation event than to the defoliation effect *per se*. These results are also consistent with the fact that the model including climate × period interaction was the best for Maritime pine: both pine species follow a similar radial growth pattern (Figure 3), led by climate characteristics.

The susceptible or host chestnut presented a moderate growth reduction (43%, Figure 3) which might be mainly attributed to the climatic characteristics, since the defoliation effect was found to be non-significant.

All these results illustrate that the differences between susceptible (radiata pine, chestnut) and resistant (Maritime pine) species to gypsy moth defoliation cannot be straight-forwardly explained in terms of differences in the pattern of radial growth as quantified by BAI, highlighting the difficulty of reconstructing past outbreak history solely by comparing tree-ring width series of coexisting host vs. non-host tree species. Outbreak tree-ring signals, as the sharp increase in minimum wood density observed in radiata pine, should be used as complementary information to tree-ring width data (Paritsis et al., 2009).

Several types of interactions between climate and insect defoliations can be discussed in this case study. First, it is possible that the particular warm and dry climatic conditions during 2011-2013 favored the gypsy moth outbreak. It is well known that spring temperature is an important factor influencing the temporal course of defoliator outbreaks, mainly due to its effects on herbivore survival and plant–herbivore synchrony (Fitzgerald, 1995). Specifically for gypsy moth, dry and warm weather in winter and spring seems to support outbreaks (Alalouni et al., 2013). However, variation in climate is not the only factor to consider since the population size of herbivorous insects must be increasing gradually prior to the outbreak, and host plants may show a progressive loss in vigor due to stressful climatic conditions (Ayres, 1993). Nevertheless, recent meta-analysis have shown that drought-stressed host plants show variable responses to herbivorous insects (Jactel et al., 2012), and therefore the subject remains under debate. Second, these particular climate conditions also contributed to reduce radial growth in non-defoliated species as Maritime pine, which growth is enhanced by wet-cool conditions during the prior autumn and winter (e.g., February) seasons (Caminero et al., 2018). Maritime pine is a plastic tree species regarding its growth responses to Mediterranean climate (Sánchez-Salguero et al., 2018), characterized by a high year-to-year variability in precipitation, which is consistent with the growth reductions observed in 2009 and 2012 in response to adverse climatic conditions. Dry and warm conditions in previous autumn and winter may reduce carbohydrate reserves by increasing respiration before the onset of tree-ring formation in spring (Kagawa et al., 2006).

In radiata pine the growth reduction started in 2012, and growth decline was irreversible in defoliated trees. Indeed, the mortality rate of severely defoliated trees of radiata pine reached in some areas values of 87% (Castedo-Dorado et al., 2016), as confirmed the last rings observed (2011-2012) in many wood samples of defoliated trees.

The absence of defoliation effect in the radial growth of chestnut trees can be explained by rapid coppicing by defoliated trees of chestnut in summer. These trees used reserved photoassimilates to rebuild their foliage and recover growth levels after the outbreak (Palacio et al., 2012). The growth pattern observed in chestnut agrees with previous studies on gypsy moth outbreaks affecting hardwood forests in north-eastern USA. There, gypsy moth caused a loss in radial growth of host oak species proportional to the defoliation severity during the year of defoliation and, in a lower degree, during the following year, whilst plot-level defoliation positively influenced the radial increment of non-host species (e.g., ash) which benefitted from increased radiation and nutrients after the death of severely defoliated trees (Muzika and Liebhold, 1999). This potential benefit for non-host species could explain the rapid increase of growth observed in non-defoliated trees of Maritime pine in 2013, whilst defoliated trees of chestnut rapidly recovered growth rates and even surpassed non-defoliated tree of chestnut in 2015.

The abundant latewood IADFs observed in 2006 and 2009 confirmed the growth sensitivity of radiata pine to the intra-annual variability in climate of the study area. Probably, wet June to July conditions favored the radial expansion of latewood tracheids leading to an increase in hydraulic conductivity and the formation of cells with wide lumen and possibly thin walls (Vieira et al., 2010). In Aleppo pine (*Pinus halepensis* Mill.) and Stone pine (*Pinus pinea* L.) latewood IADFs were also formed in response to humid conditions in summer and autumn when cell-wall thickening and lignification are active phases of xylogenesis (Campelo et al., 2007; Pacheco et al., 2018). The IADFs did not translate into enhanced radial growth but the formation of earlywood-like tracheids within the latewood probably explained the reduction in maximum wood density in combination with warmer temperature in autumn.

One of the most striking results of this study was the increase of minimum wood density of radiata pine in response to the 2012 defoliation. Planted Maritime pine subjected to induced defoliation by *Thaumetopoea pityocampa* Den. & Schiff. showed a ca. 30% reduction in tree-ring width but no differences in maximum wood density when compared with non-defoliated trees (Polge and Garros, 1971). However, the minimum wood density of the defoliated trees decreased the defoliation year, but increased two years later. In planted Norway spruce (*Picea abies* L.), outbreaks of nun moth (*Lymantria monacha* L.) were associated with the production of narrow rings with higher minimum wood density (Koprowski and Duncker, 2012). Most studies using tree-ring data to reconstruct or to quantify the impacts of population outbreaks of forest Lepidoptera have documented reductions of radial growth associated with severe defoliations (Swetnam and Lynch, 1993; Krause, 1997; Weber, 1997; Rolland et al., 2001; Speer et al., 2001; Camarero et al., 2003; Vejpustková and Holuša, 2006; Sutton and Tardif, 2007; Sangüesa-Barreda et al., 2014), and sometimes a decrease in maximum wood density reflecting a reduction in the thickening and lignification rates of latewood tracheids (Esper et al., 2007). The finding that gypsy moth defoliation increased minimum wood density may be explained considering xylogenesis processes and its climate drivers. In planted radiata pine stands, wet conditions are associated with improved growth, whereas warm conditions during the growing season are associated with a reduction in wood density (Ivković et al., 2013). Minimum wood density is positively associated with spring temperatures, prior winter precipitation and drought severity in plantations in Australia and New Zealand (Harris, 1965; Downes et al., 1993). This indicates that warmer spring conditions favor photosynthesis and increase wood density by increasing wall thickness, whereas drought stress will increase density by decreasing the radial diameter of tracheids. In fact, drought was associated with increases in minimum wood density of planted radiata pines (Downes et al., 1993). In this case defoliation probably altered the water use within trees, reduced the turgour of cambial derivatives and induced the formation of earlywood tracheids with narrow lumen (Skene, 1969) leading to an increase in minimum wood density as has been observed in other conifers under natural conditions (Camarero et al., 2014). To disentangle the defoliation from the climate effects on wood formation, further studies could quantify density changes in non-host species as Maritime pine as compared with host species as radiata pine. Regarding maximum wood density in radiata pine, it has been found to be negatively associated with severe stress due to summer drought (Downes et al., 1993), in our case this could correspond to warm September conditions amplifying late-summer drought.

Interestingly, defoliated trees of radiata pine showed lower growth and maximum and minimum wood density values than non-defoliated trees during the 2000–2011 period, i.e., prior to the 2012–2013 outbreak. According to the growth-defense trade-off hypothesis (Herms and Mattson, 1992), we would expect that trees showing low growth rates can invest more on defenses and therefore are less susceptible to defoliation, whilst fast-growing trees would be more susceptible to defoliation. In this case slow-growing radiata pines were also more defoliated, not supporting that trade-off at the within-species level. Differences in radial growth cannot be attributed either to microsite differences since BAI previous to defoliation and during the defoliation was not found to be spatially autocorrelated; i.e., the spatial distribution of BAI is likely to be the result of random spatial processes.

In chestnut, BAI of defoliated trees was also lower than that of non-defoliated trees from 2009 to 2011. Remarkably, chestnut trees show lower grow rates and may be genetically predisposed to invest more in defense than fast-growing radiata trees according to the growth-defense trade-off hypothesis applied at the between-species level. Probably as a consequence of greater amounts of defense in chestnut, the impact of defoliation has been negligible. Nevertheless, we note that growth-defense trade-offs are complex to disentangle within and between species and may be attenuated under favorable climate conditions (Züst and Agrawal, 2017).

All these results confirm that the most defoliated trees, particularly in the case of radiata pine, were previously growing less and forming less dense wood than trees less affected by defoliation. This could mean that they were predisposed to show more pronounced gypsy-moth defoliation and they were more prone to die than fast-growing trees as has been observed in drought-induced forest dieback studies in natural pine populations (Camarero et al., 2015). Regrettably, the radiata pines used for obtaining BAI and density were not the same. Therefore, future studies could concentrate on evaluating the relationships between BAI and wood density in defoliated trees. The 2012 increase in minimum wood density, possibly reflecting a reduction in the transversal area of earlywood tracheids, was coherent with the reduction in growth since earlywood accounts for most of the hydraulic conductivity within the ring (Domec et al., 2009).

## Conclusions

Abnormal warm and dry conditions in the winter prior to a gypsy moth outbreak reduced radial growth in both host (radiata pine and chestnut) and non-host (Maritime pine) species and might have resulted in the defoliation event occurred in 2012–2013.

Defoliation induced an increase in minimum wood density of radiata pine, probably reflecting a reduction in earlywood hydraulic conductivity and radial-growth rates. Therefore, this complementary information to tree-ring width data must be used in reconstructing past outbreak history of coexisting host vs. non-host tree species.

Radiata pine plantations could show relevant losses in productivity and changes in wood density due to potential interactions between climate warming and gypsy-moth outbreaks.

## Author contributions

JC, FA-T, and FC-D conceived the idea. AH performed and interpreted the densitometry analyses. All authors contributed to the field work and the interpretation of the results. The dataset was analyzed by JC, FC-D, and FA-T. JC and FC-D wrote the first draft of the manuscript; thereafter all authors revised the first draft by rewriting, discussing and commenting. All authors read and approved the final draft.

### Conflict of interest statement

The authors declare that the research was conducted in the absence of any commercial or financial relationships that could be construed as a potential conflict of interest.
