# Supplementary material for: Radial Growth and Wood Density Reflect the Impacts and Susceptibility to Defoliation by Gypsy Moth and Climate in Radiata Pine
Source: Front Plant Sci. 2018 Oct 31;9:1582. doi: 10.3389/fpls.2018.01582 (PMC6220356; doi:10.3389/fpls.2018.01582)
Supplement: Supplementary file 1 [file Table_1.docx]

**Supporting Information**

(a)

**
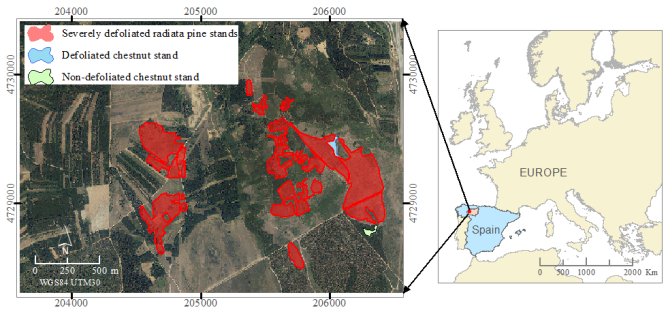

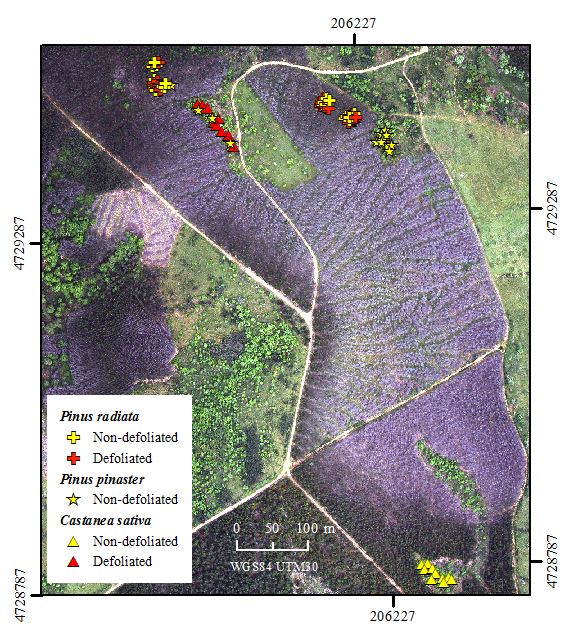
**

(b)

**Figure S1.** (a) Climate diagram of the Ponferrada station, situated near the study area; and (b) location of the sampled trees in north-western Spain (see the inset) showing the situation of: defoliated and non-defoliated radiata pine (*Pinus radiata*) trees, defoliated and non-defoliated chestnut (*Castanea sativa*) trees, and non-defoliated Maritime pine (*Pinus pinaster*) trees.

**Figure S2.** Climate trends at the Ponferrada station based on seasonal data (different symbols and colors indicate different seasons). Variables with linear regressions show significant trends (*P* < 0.05) for the 1971-2015 period according to the Kendall τ statistic. The grey boxes indicate the gypsy moth outbreak period.

**Figure S3.** Climatic anomalies (values subtracted from the mean and divided by the standard deviation) for monthly variables (mean maximum and minimum temperatures, precipitation) considering selected years according to radiata pine radial growth. Climate data correspond to the Ponferrada station and the 1971-2015 period. Selected years correspond to narrow rings (2005 and 2006), abundant production of intra-annual density fluctuations in the latewood (2009) and the starting (2011) and peak of the gypsy outbreak (2012-2013).

**Figure S4.** Ring-width indices of the three studied tree species and shown for defoliated and non-defoliated trees. The gypsy moth outbreak caused defoliation of chestnut trees and radiata pine trees but it did not affect Maritime pine. Values are means ± SE.

|  |  |
| --- | --- |

**Figure S5.** Non-climatic BAI residuals (difference between observed BAI and the predicted values by the linear mixed models) for *Pinus radiata* (left) and *Castanea sativa* (right) considering the outbreak period (2012-2013). Values are means ± SE.

**Table S1.** Analyses of the temporal autocorrelation of monthly climate variables (mean of the maximum and mean of the minimum temperatures, precipitation). Climate data were taken from the Ponferrada station. No significant (*P* < 0.05) autocorrelation coefficients were detected. The analyses were carried out up to 7 lags considering the period 2000-2015.

| Lag (years) | Maximum temperature | | | | | | | | | | | |  |
| --- | --- | --- | --- | --- | --- | --- | --- | --- | --- | --- | --- | --- | --- |
|  | Jan | Feb | Mar | Apr | May | Jun | Jul | Aug | Sep | Oct | Nov | Dec | |
| 1 | -0.09 | 0.19 | -0.54 | -0.03 | -0.35 | 0.34 | -0.14 | -0.41 | 0.11 | 0.02 | -0.02 | -0.33 | |
| 2 | -0.55 | -0.08 | -0.14 | -0.52 | -0.20 | -0.02 | -0.07 | 0.03 | 0.28 | 0.42 | -0.03 | -0.11 | |
| 3 | 0.14 | -0.18 | 0.59 | 0.06 | 0.18 | -0.16 | 0.06 | -0.09 | -0.06 | 0.22 | -0.19 | 0.23 | |
| 4 | -0.22 | 0.02 | -0.42 | 0.50 | -0.23 | -0.10 | -0.03 | 0.15 | 0.04 | 0.12 | 0.25 | 0.09 | |
| 5 | -0.01 | -0.32 | -0.15 | 0.15 | 0.19 | 0.24 | 0.12 | -0.16 | -0.40 | 0.07 | -0.21 | -0.54 | |
| 6 | 0.61 | -0.52 | 0.26 | -0.55 | 0.59 | 0.00 | -0.40 | -0.20 | 0.30 | -0.08 | -0.15 | -0.04 | |
| 7 | -0.01 | -0.20 | 0.27 | -0.19 | -0.80 | -0.48 | 0.26 | 0.38 | -0.13 | -0.08 | -0.47 | 0.32 | |
| Minimum temperature | | | | | | | | | | | | | |
| 1 | -0.28 | 0.25 | -0.01 | 0.13 | 0.12 | 0.38 | 0.00 | -0.12 | -0.04 | 0.05 | -0.20 | -0.52 | |
| 2 | -0.40 | -0.42 | 0.30 | -0.32 | -0.55 | -0.14 | -0.08 | 0.08 | -0.02 | -0.39 | -0.45 | 0.05 | |
| 3 | 0.29 | -0.19 | -0.02 | 0.23 | -0.07 | -0.07 | -0.16 | -0.43 | 0.22 | 0.01 | 0.57 | 0.27 | |
| 4 | -0.41 | -0.31 | -0.33 | 0.50 | -0.06 | -0.19 | 0.13 | -0.24 | 0.21 | 0.19 | 0.00 | -0.18 | |
| 5 | 0.01 | -0.39 | 0.51 | 0.21 | -0.04 | 0.31 | 0.25 | 0.05 | -0.34 | 0.08 | -0.25 | -0.46 | |
| 6 | 0.51 | 0.28 | -0.55 | -0.36 | 0.07 | 0.69 | -0.39 | 0.49 | -0.26 | -0.80 | 0.36 | 0.28 | |
| 7 | 0.14 | 0.74 | 0.00 | 0.05 | -0.23 | -0.17 | 0.07 | 0.10 | 0.11 | -0.18 | -0.22 | -0.27 | |
| Precipitation | | | | | | | | | | | | | |
| 1 | -0.23 | 0.40 | -0.29 | 0.03 | -0.20 | 0.41 | -0.09 | -0.36 | 0.24 | -0.18 | -0.13 | -0.53 | |
| 2 | -0.50 | -0.15 | -0.20 | -0.58 | -0.39 | -0.08 | -0.06 | 0.10 | 0.02 | -0.04 | -0.50 | 0.08 | |
| 3 | 0.23 | -0.47 | 0.57 | 0.13 | 0.19 | -0.13 | -0.02 | -0.23 | 0.32 | 0.10 | 0.47 | 0.39 | |
| 4 | -0.35 | -0.35 | -0.44 | 0.66 | -0.22 | -0.08 | 0.05 | 0.05 | -0.02 | 0.20 | 0.20 | -0.09 | |
| 5 | 0.00 | -0.28 | -0.39 | 0.18 | 0.24 | 0.28 | 0.15 | -0.15 | -0.22 | 0.50 | -0.18 | -0.64 | |
| 6 | 0.70 | -0.05 | 0.58 | -0.56 | 0.52 | 0.30 | -0.42 | 0.05 | -0.10 | -0.55 | 0.13 | 0.17 | |
| 7 | 0.18 | 0.26 | -0.45 | -0.22 | -0.72 | -0.43 | 0.22 | 0.33 | 0.65 | 0.18 | -0.12 | -0.14 | |

**Table S2.** Parameter estimates and their significance for the nested mixed effects models analyzed. Note: *** = significant at 0.001 significance level; ** = significant at 0.05 significance level. Selected models for radiata pine and Maritime pine are highlighted in bold.

| *Pinus radiata* | | | | | | | | | |
| --- | --- | --- | --- | --- | --- | --- | --- | --- | --- |
| Number of model interactions | *b*_0_ | *b*_1_  (Tmin autumn*_t_*_-1_) | *b*_2_  (Defoliation) | *b*_3_  (Period) | *b*_12_  (Tmin autumn*_t_*_-1_ × Defoliation) | *b*_13_  (Tmin autumn*_t_*_-1_ × Period) | *b*_23_  (Defoliation × Period) | *b*_123_  (Tmin autumn*_t_*_-1_ × Defoliation × Period) | **Log lik** |
| 1 three-way interaction | 36.1  *** | -3.73  *** | -9.63 | -29.60  *** | 1.16 | 3.79  *** | 9.20 | -1.15 | -314.0 |
| 3 two-way interactions | 34.6  *** | -3.55  *** | -0.505  ** | -28.1  *** | 0.00929 | 3.60  *** | 0.0681 | - | -314.1 |
| 2 two-way interactions | 34.7  *** | -3.56  *** | -0.451  ** | -28.2  *** | 0.00889 | 3.62  *** | - | - | -314.3 |
| 2 two-way interactions | 6.25  *** | 0.0568  *** | -0.590  ** | 0.299  *** | 0.0100 | - | 0.0927 | - | -339.4 |
| 2 two-way interactions | 34.6  *** | -3.55  *** | -0.432  ** | -28.1  *** | - | 3.61  *** | 0.0657 | - | -314.3 |
| 1 single interaction | 6.23  *** | 0.0569  *** | -0.514  ** | 0.328  *** | 0.00947 | - | - | - | -327.5 |
| **1 single interaction** | **34.7**  ******* | **-3.56**  ******* | **-0.382**  ****** | **-28.3**  ******* | **-** | **3.62**  ******* | **-** | **-** | **-314.4** |
| 1 single interaction | 6.22  *** | 0.0605  *** | -0.510  ** | 0.300  *** | - | - | 0.0913 | - | -327.4 |
| *Pinus pinaster* | | | | | | | | | |
| Number of model interactions | *b*_0_ | *b*_1_  (Tmin autumn*_t_*_-1_) | *b*_2_  (Period) | *b*_12_  (Tmin autumn*_t_*_-1_ × Period) |  | | | | **Log lik** |
| **1 single interaction** | **37.1**  ******* | **-3.81**  ******* | **-30.4**  ******* | **3.88**  ******* |  |  |  |  | **-56.0** |
| None | 6.60  *** | 0.0700  *** | 0.254  *** | - |  |  |  |  | -65.5 |
| *Castanea sativa* | | | | | | | | | |
| Number of model interactions | *b*_0_ | *b*_1_  (Tmax spring) | *b*_2_  (Defoliation) | *b*_3_  (Period) | *b*_12_  (Tmax spring × Defoliation) | *b*_13_  (Tmax spring × Period) | *b*_23_  (Defoliation × Period) | *b*_123_  (Tmax spring × Defoliation × Period) | **Log lik** |
| 1 three-way interaction | 6.03  *** | -0.00707  * | -0.797 | -2.34 | 0.0289 | 0.120 | 1.90 | -0.0825 | -211.0 |
| 3 two-way interactions | 5.38  *** | 0.0285  * | 0.453 | -1.50 | -0.0387 | 0.0764 | 0.299 | - | -211.4 |
| 2 two-way interactions | 5.45  *** | 0.0188  ** | 0.297 | -1.32 | -0.0200 | 0.0757 | - | - | -212.2 |
| 2 two-way interactions | 4.21  *** | 0.0904  *** | 0.432 | -0.00622 | -0.0377 | **-** | 0.294 | - | -212.5 |
| 2 two-way interactions | 5.71  *** | 0.00807  ** | -0.185 | -1.43 | - | 0.0757 | 0.209 | - | -211.9 |
| 1 one-way interaction | 4.30  *** | 0.0804  *** | 0.277 | 0.149 | -0.0193 | **-** |  | - | -213.3 |
| 1 one-way interaction | 5.64  *** | 0.00823  ** | -0.0614 | -1.32 | - | 0.0754 | - | - | -213.3 |
| 1 one-way interaction | 4.56  ******* | 0.0700  ******* | -0.189 | 0.0407 | - | - | 0.208 | - | -212.0 |
